# Risk Factor Analysis of *Mycoplasma pneumoniae* Pneumonia Complicated With Plastic Bronchitis in Children: A Single‐Center Retrospective Study

**DOI:** 10.1111/crj.70142

**Published:** 2025-12-04

**Authors:** Jian‐Min Dong, Chun‐Qing Zhou, Yuan‐Yuan Zhen

**Affiliations:** ^1^ Department of Pediatrics Feicheng People's Hospital Feicheng China; ^2^ Department of Pediatrics Qingdao Hiser Hospital Affiliated with Qingdao University (Qingdao Traditional Chinese Medicine Hospital) Qingdao China; ^3^ Department of Pediatrics Qilu Hospital of Shandong University (Qingdao) Qingdao China

**Keywords:** bronchitis, child group, *Mycoplasma*, pneumonia, regression analysis, risk factors

## Abstract

**Objective:**

To investigate the clinical features and risk factors for *Mycoplasma pneumoniae* pneumonia (MPP) complicated with plastic bronchitis (PB) and to provide a reference for the early diagnosis and treatment of this disease.

**Methods:**

Clinical data from 75 pediatric patients diagnosed with MPP who underwent bronchoscopy at our hospital between June 16 and December 31, 2023, were retrospectively analyzed. Patients were stratified into PB and non‐PB groups based on the presence or absence of bronchial cast removal during bronchoscopy. Comparative analysis of clinical characteristics was performed between the two groups. Binary logistic regression analysis was employed to identify risk factors associated with MPP complicated by PB. Additionally, bronchial cast components obtained from the PB group underwent compositional analysis using proteomic techniques via mass spectrometry.

**Results:**

The composition ratio of children with fever frequency and a heat course ≥ 10 days in the PB group. The composition ratio, neutrophil ratio, erythrocyte sedimentation rate (ESR) and levels of C‐reactive protein, lactate dehydrogenase, procalcitonin (PCT) and D‐dimer in children with lung compaction were significantly greater than those in the non‐PB group (*t* = 2.290–3.793, χ^2^ = 5.548, 5.659, *Z* = −2.085, *p* < 0.05). Multivariate logistic regression analysis revealed that the PCT level (OR = 1.071, 95% CI = 1.015–1.130, *p* < 0.05) and ESR (OR = 1.088, 95% CI = 1.033–1.146, *p* < 0.05) were risk factors for PB. Protein mass spectrometry showed that the bronchial plastic was rich in fibrinogen.

**Conclusions:**

Compared with children with MPP alone, children with PB have a more intense inflammatory response, and the possibility of MPP with PB should be vigilant when the ESR > 25.20 mm/1 h and PCT > 0.19 μg/L. Bronchial plastics in children with PB contain a large amount of fibrin, which may be related to the abnormal activation of coagulation and fibrinolysis systems caused by inflammation.

## Graphical abstract

Risk factor prognostic model analysis of *Mycoplasma pneumoniae* pneumonia in children: a single‐center retrospective study.

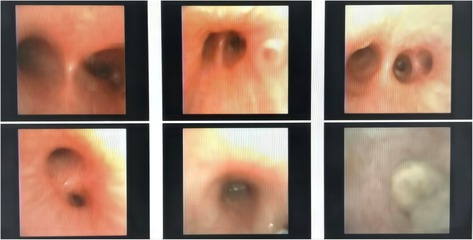

## 1 Introduction

Plastic bronchitis (PB) is an acute, critical respiratory disorder characterized by the formation of densely branched, gelatinous casts within the tracheobronchial tree. These fibrin‐rich casts, which vary in size and morphology, result from multifactorial pathogenic mechanisms [1, 2, 3]. As a clinically rare entity, the precise pathogenesis of PB remains incompletely elucidated. Due to variations in the extent and severity of airway obstruction, PB induces respiratory dysfunction characterized by impaired ventilation and gas exchange. This manifests clinically as a spectrum of symptoms including cough, wheezing, fever, chest tightness, and chest pain [4]. In severe cases, patients may develop acute respiratory failure with life‐threatening implications [5]. Importantly, *Mycoplasma pneumoniae* infection represents a predominant predisposing factor for PB. Notably, PB can occur even in children exhibiting only mild infectious symptoms. With the expanding clinical application of bronchoscopy, reported cases of MP‐associated PB have shown a progressive increase [6, 7].

*Mycoplasma pneumoniae* (MP) represents a predominant etiological agent of community‐acquired pneumonia (CAP) in children, manifesting as a spectrum of respiratory disease ranging from mild upper respiratory tract infections to severe pneumonia [8]. Recent advances in understanding MP pathogenesis have heightened clinical recognition of its extrapulmonary complications. Among these, PB constitutes a critical complication. Despite its relatively low incidence, PB poses a substantial health burden in the pediatric population [9]. Although prior investigations have examined the pathological mechanisms and therapeutic approaches for PB, a notable gap persists in systematic analyses delineating the clinical characteristics and risk factors for PB secondary to *M. pneumoniae* pneumonia (MPP).

In this study, the clinical characteristics and risk factors for children with MPP complicated with PB were investigated. Through a systematic review and analysis of the clinical data of the children, the clinical manifestations, laboratory examinations, imaging characteristics and treatment effects were summarized, and the possible risk factors were further discussed. Through the analysis of these factors, we hope to provide a valuable reference for clinicians to identify and intervene early in clinical practice, reduce the incidence of complications, and improve the prognosis of children.

## 2 Data and Methods

### 2.1 Study Design and Participants

Children diagnosed with MPP and who underwent bronchoscopy in the Department of Pediatrics of Qilu Hospital of Shandong University (Qingdao) between July 16 and December 31, 2023, were selected for this study. According to whether the plastic was removed under bronchoscopy, the patients were divided into a PB group and a non‐PB group.

The inclusion criteria for patients were as follows: (1) met the diagnostic criteria for MPP in the Expert Consensus on Diagnosis and Treatment of MPP in Children [10], requiring fulfillment of all the following criteria: (i) primary clinical manifestations of fever and cough, so as to ensure the case was clinically infected rather than asymptomatic carriers; (ii) serological confirmation (single serum MP antibody titer ≥ 1:160 via particle agglutination [PA] assay) or positive MP‐DNA/RNA detection; (iii) radiographically verified pulmonary inflammation on chest x‐ray or CT. (2) underwent bronchoscopy in accordance with the Chinese Pediatric Flexible Bronchoscopy Guidelines; (3) were ≤ 6 months of age and ≤ 14 years of age, with a disease course before admission of 5–30 days; (4) signed informed consent for bronchoscopy; and (5) Following a standardized 5‐ to 7‐day course of macrolide antibiotic therapy, pediatric patients who exhibited persistent high fever alongside unimproved or worsening clinical symptoms, including cough and shortness of breath, or manifested severe wheezing, dyspnea, and tachypnea which remained unresponsive to conventional interventions such as oxygen therapy and nebulization with bronchodilators, necessitated consideration of potential airway obstruction by mucus plugs or inflammatory exudates.

The exclusion criteria were as follows: (1) had severe arrhythmia, bleeding disease, coagulation dysfunction, cardiopulmonary or liver and kidney insufficiency, congenital immune deficiency, or congenital heart disease; (2) had bacterial pneumonia, viral pneumonia, tuberculosis, bronchial foreign body, or other respiratory diseases; (3) were allergic to therapeutic drugs or had poor compliance; and (4) had contraindications for bronchoscopy.

A total of 75 children were ultimately included in the study, including 21 in the PB group and 54 in the non‐PB group. There was no significant difference in the general data between the two groups (Table 1).

**TABLE 1:** Comparison of general information between two groups of children.

Index	PB group (*n* = 21)	Non‐PB group (*n* = 54)	*Z*/*t*/χ^2^	*p*
Gender (male/female)	10/11	25/29	0.011	0.918
Age	6.33 ± 1.82	5.87 ± 2.13	0.875	0.384
Children aged ≥ 6 years old [(%)]	14(66.7)	31(57.4)	0.132	0.717
BMI (kg/m^2^)	15.41 ± 1.87	15.29 ± 2.35	0.209	0.835

### 2.2 Data Collection

(1) Heat indices are as follows: heat course, heat peak, heat frequency; (2) laboratory test indicators are as follows: white blood cell count, neutrophil count, platelet count, pulmonary alveolar lavage fluid (BALF) cell classification test, erythrocytosis decline rate (ESR) and C‐reactive protein (CRP), lactate dehydrogenase (LDH), alkaline phosphatase (ALP), procalcitonin (PCT), D‐dimer, and alanine aminotransferase (ALT) levels; (3) results of chest CT examination; (4) bronchoscopic manifestations; (5) protein mass spectrometry analysis of bronchial plastic.

### 2.3 Data Processing

SPSS 25.0 software was used for analysis. Normally distributed data are shown as x̄ ± SD, and *t* test was used for intergroup comparisons. The measured materials that did not conform to a normal distribution are expressed as M (P25, P75), and comparisons between groups were performed using nonparametric tests. The statistical data are expressed as rates. The χ^2^ test, Pearson χ^2^ test or continuously adjusted χ^2^ test was used for comparisons between groups. Binary logistic regression analysis was performed to determine significant differences between the two groups. *p* < 0.05 was considered to indicate statistical significance.

## 3 Results

### 3.1 Comparison of Febrile Manifestations and Chest CT Results Between the Two Groups

A comparative analysis was conducted on the fever manifestations and chest CT examination results of two groups of pediatric patients. Regarding the duration of fever, in the PB group, 61.90% (13 cases) of patients had a fever lasting for 10 days or more, whereas in the non‐PB group, only 27.78% (15 cases) of patients had a fever duration of 10 days or more. A statistically significant difference was observed between the two groups (*p* < 0.01). In terms of the fever frequency, the mean daily fever frequency in the PB group was 3.33 ± 1.85 times, significantly higher than that in the non‐PB group, which was 2.31 ± 1.17 times per day (*p* < 0.01). Chest CT examination results revealed that 33.33% of patients in the PB group presented with pulmonary consolidation, while the proportion in the non‐PB group was merely 7.41%, and this difference was statistically significant (*p* < 0.05). However, there were no statistically significant differences in peak fever, atelectasis, and pleural effusion between the two groups (Table 2).

**TABLE 2:** Comparison of fever manifestations and chest CT examination results between two groups of pediatric patients.

Index	PB group (*n* = 21)	Non‐PB group (*n* = 54)	*Z*/*t*/χ^2^	*p*
Thermal process (%)				
<10 d	8 (38.1)	39 (72.2)	7.527	**0.006**
≥10 d	13 (61.9)	15 (27.8)		
Heat peak (t/°C)	39.28 ± 0.97	39.16 + 0.77	0.539	0.595
Fever frequency (times/d)	3.33 ± 1.85	2.31 ± 1.17	2.836	**0.006**
Pulmonary consolidation (%)	7 (33.3)	4 (7.4)	5.548	**0.018**
Atelectasis (%)	2 (9.5)	6 (11.1)	0.032	0.857
Pleural effusion (%)	5 (23.8)	11 (20.4)	0.068	0.794

*Note:* Count data are expressed as rates (%), and differences are compared using the chi‐square test. Measurement data that conform to a normal distribution are expressed as mean ± standard deviation, and differences are compared using the *t* test. Measurement data that do not conform to a normal distribution are expressed as median (P25, P75), and differences are compared using the rank‐sum test. *p* < 0.05 was considered to indicate statistical significance.

### 3.2 Comparison of Laboratory Examination Results Between the two Groups

As presented in Table 3, this study further compared the laboratory test results between pediatric patients in the PB group and those in the non‐PB group. In the context of BALF cell classification, the median proportion of neutrophils in the PB group was 62 (44, 73), significantly higher than the 47 (33, 66) in the non‐PB group(*p* < 0.05). The PB group exhibited an ESR of 35.09 ± 10.02 mm/h, while the non‐PB group had 23.20 ± 12.90 mm/h, with these differences reaching statistical significance (*p* < 0.001). The level of CRP in the PB group was approximately 2.9 times that of the non‐PB group; the LDH level was about 1.35 times higher, and the expression of PCT was 2.18 times that in the non‐PB group. As a specific marker for secondary hyperfibrinolysis, the D‐dimer levels in the PB group were significantly elevated compared to those in the non‐PB group (*p* < 0.01). Conversely, several indicators including white blood cell count, neutrophil count, platelet count, the proportions of macrophages, lymphocytes, and eosinophils in BALF, along with ALT and ALP, did not show statistically significant differences between the two groups, suggesting that these indicators have limited utility in differentiating pediatric patients between the PB and non‐PB groups.

**TABLE 3:** Comparison of laboratory test results between two groups of pediatric patients.

Index	PB group (*n* = 21)	Non‐PB group (*n* = 54)	*t*/*Z*	*p*
White blood cell count (×10^9^/L)	6.77 ± 2.94	7.73 ± 2.88	−1.281	0.204
Neutrophil count (×10^9^/L)	4.72 ± 2.32	5.31 ± 6.70	−0.386	0.70
Platelet count (×10^9^/L)	309.76 ± 115.50	328.98 ± 94.21	−0.744	0.459
**BALF cell classification detection**				
Macrophages (%)	27 (14, 43)	35 (24, 55)	−1.446	0.148
Neutrophils (%)	62 (44.73)	47 (33, 66)	−2.085	**0.037**
Lymphocytes (%)	10 (3.15)	5 (2, 19)	−0.314	0.753
Eosinophils (%)	0 (0, 2)	0 (0, 1)	−0.997	0.329
ESR (mm/h)	35.09 ± 10.02	23.20 ± 12.90	3.793	**<0.001**
CRP level (mg/L)	50.56 (32.32, 69.84)	17.27 (10.98, 30.17)	−4.106	**<0.001**
LDH level (U/L)	407.85 ± 199.04	301.73 ± 94.36	2.343	**0.028**
PCT level (μg/L)	0.31 (0.21, 0.44)	0.13 (0.09, 0.17)	−4.724	**<0.001**
D‐dimer level (μg/L)	2509 (1627,3892)	952 (6161392)	−4.519	**<0.001**
ALT level (U/L)	27.51 (21.56, 35.01)	31.93 (19.24, 51.93)	−1.121	0.262
ALP level (U/L)	122.80 ± 37.88	129.99 ± 31.34	−0.836	0.406

*Note:* Measurement data that conform to a normal distribution are expressed as mean ± standard deviation, and differences are compared using the *t* test; measurement data that do not conform to a normal distribution are expressed as median (P25, P75), and differences are compared using the rank‐sum test. *p* < 0.05 was considered to indicate statistical significance.

### 3.3 Bronchoscopy Results of the Two Groups of Children

**FIGURE 1:**
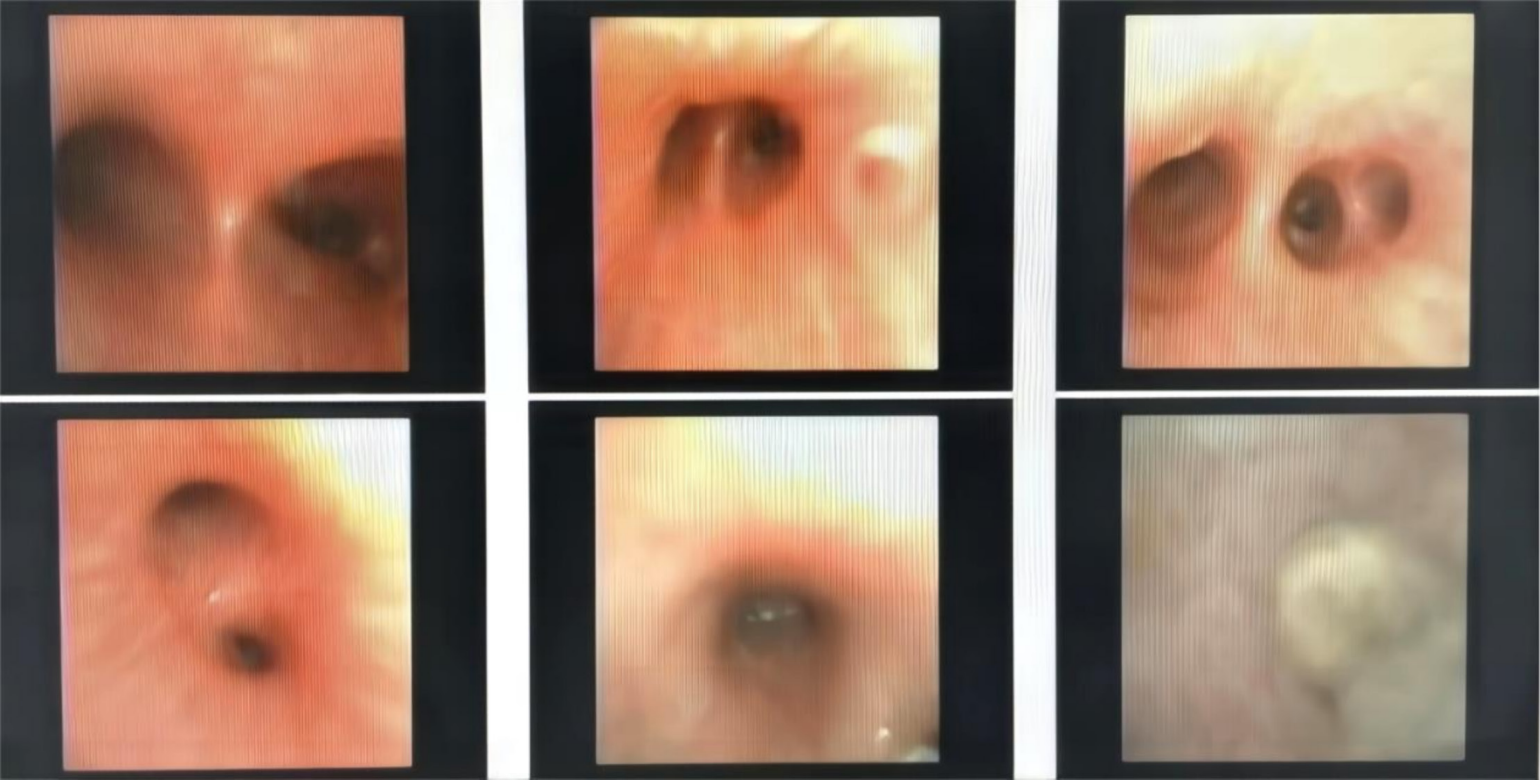
Pulmonary bronchoscopic findings in the PB group.

In the PB group, the left lower lung was the most frequently affected site of obstruction. When examined under the branch bronchoscope, the bronchial mucosa exhibited congestion, edema, and chylous alterations, along with wrinkling and thickening of the bronchial wall. Numbers of bronchial orifices presented with different degrees of inflammatory edema and stenosis. Concurrently, there was an increase in viscous secretions within the bronchial lumen, and grayish‐colored secretions were noted in the obstructed regions. In this group, it was possible to extract a branch‐shaped, jelly‐like bronchial cast, measuring approximately 2–5 cm in length. This cast was characterized by high elasticity and remarkable resistance to breakage. In contrast, in the non‐PB group, bronchoscopy merely detected varying degrees of hyperemia and edema of the bronchial mucosa (Figure 1).

### 3.4 Identification of Independent Risk Factors for MPP Complicated With PB

The heat course (<10 d = 0, ≥10 d = 1), lung consolidation (no = 0, yes = 1), fever frequency (no = 0, yes = 1), BALF neutrophil ratio, ESR and CRP, LDH, PCT and D‐dimer levels were considered independent variables, and concurrent PB was considered the dependent variable (no = 0, yes = 1). Binary multivariate logistic regression analysis was performed, and the results showed that PCT level (OR = 1.071, 95% CI = 1.015–1.130, *p* < 0.05) and ESR (OR = 1.088, 95% CI = 1.033–1.146, *p* < 0.05) were found to be independent risk factors for MPP complicated with PB. See Table 4.

**TABLE 4:** Multivariate logistic regression analysis of concurrent PB in MPP.

Index	*B*	SE	Wald value	*p*	OR value	95% CI
PCT level	0.069	0.027	6.230	0.013	1.071	1.015–1.130
ESR	0.084	0.027	9.997	0.002	1.088	1.033–1.146

### 3.5 Protein Mass Spectrometry Analysis of Bronchial Plastic in the PB Group

The results of protein mass spectrometry of bronchial plastica in the PB group showed that the bronchial plastica of the PB group contained abundant fibrinogen (α, β, γ chain) and a small amount of MUC5B and MUC5AC. In addition, abundant immune cell proteins (immunoglobulin γ, α, and μ) and proteases were detected.

## 4 Discussion

At present, the incidence of MPP in children in China is increasing annually. In some children with MPP, the disease progresses rapidly, with irritating cough in the early stage and excessive phlegm in the later stage, which makes it difficult for them to cough. Bronchoscopy shows that there is a focal or extensive mucous increase in the trachea, or secretions are blocked to form tubes, and branch‐like bronchial plastic can be extracted. This study analyzed the clinical features and risk factors for PB in children with MPP, aiming to provide a basis for the early diagnosis and treatment of PB [11].

A total of 75 children were enrolled in this study, among whom 21 had concomitant PB, with an incidence rate of 28%. This rate was significantly higher than that reported in other regions of the province [6] while similar to the findings reported by Ma et al. [12]. Previous literature has reported that PB is high in children aged 4–12 years, which may be related to the lack of strong support of children's bronchial wall and the difficulty of discharge of secretions in the tube. Older children have a stronger lung tissue immune response, so PB is more likely to occur [13]. In this study, the average age of the PB group was 6.33 ± 1.82 years, and 66.6% of the participants were children aged 6 years or older, which was similar to previous studies. The children in the PB group mainly had fever and cough, and most of them had a high fever at admission. The proportions of children with a fever frequency and heat course ≥ 10 days were greater than those in the non‐PB group, which had characteristics of long disease course and difficult recovery. Meanwhile, children in the PB group exhibited a significantly elevated frequency of fever. The prolonged disease course and heightened fever frequency synergistically accelerated the release of inflammatory factors, promoted goblet cell hyperplasia, induced airway edema and stenosis, and consequently hindered the timely clearance of secretions. This cascade of events further increased the risk of bronchial plastic formation [14, 15].

Pulmonary consolidation arises from inflammatory exudation and necrotic tissue accumulation during the formation of bronchial casts [16]. In this study, 33.3% of children in the PB group exhibited lung consolidation, a proportion significantly higher than that in the non‐PB group. Additionally, 23.8% of the PB group presented with pleural effusion (all demonstrating small ipsilateral effusions), and 9.5% showed atelectasis. Multiple studies have shown that lung consolidation is a typical imaging feature of PB [2, 17, 18], which were consistent with the conclusions of this study. This further confirms the diagnostic value of lung consolidation in MPP complicated with PB.

CRP is an acute‐phase nonspecific reactive protein that appears within 12–48 h after inflammatory stimulation and is also a sensitive indicator of the systemic inflammatory response and is positively correlated with the severity of MPP [19]. LDH is also a nonspecific inflammatory marker closely related to myocardial cell damage, and its expression is low in lung tissue; however, a large amount of LDH can be released into the blood during severe lung infection. LDH is positively correlated with the severity of MPP [20]. In this study, CRP and LDH levels in the PB group were significantly greater than those in the non‐PB group, suggesting more severe pulmonary inflammatory injury in the PB group. Notwithstanding the limitations in detection conditions, this study had yet to perform a systematic comparative analysis of serum inflammatory factor levels and immune cell subset composition among the enrolled pediatric cohort, which constrained partially compromises the comprehensiveness of the derived conclusions. Prior research by Zhong et al. delineated differential distribution patterns of T cell subsets between children with PB and non‐PB groups, thereby substantiating T cell dysfunction as a potential risk factor for MPP complicated by PB. Their findings concurrently illuminate the diagnostic utility of immune cell dysregulation in MPP‐PB comorbidity [21]. Building upon this foundation, subsequent investigations should broaden the research scope to encompass phenotypic and functional alterations in pivotal immune cell subsets, including macrophages and NK cells. Such expansion would elucidate the core mechanistic underpinnings of inflammatory pathways as risk determinants for MPP‐PB progression and fortify the evidentiary basis for refining its risk stratification framework.

The ratio of BALF neutrophils in the PB group was greater than that in the non‐PB group, suggesting that there may be an active local inflammatory response when lung tissue injury occurs. A sustained inflammatory response can cause local vascular damage, destroy the integrity of vascular endothelial cells, expose subcellular collagen, activate the coagulation cascade signaling pathway, and form fibrin monomers under the action of thrombin, which then form fibrin albumin clots under FIIIa crosslinking and accumulate to form bronchial plastic [22, 23]. Notably, a latest study has pointed out that FIIIa promotes the formation and crosslinking of fibrin networks through neutrophil extracellular traps (NETs) [22, 23], suggesting the possible role of NETs in bronchial remodeling, which deserves further attention.

Fibrin clots can produce D‐dimer via the cleavage of plasminase, and D‐dimer is the main degradation marker of fibrin. In this study, the D‐dimer level was significantly different between the two groups of patients, reflecting the formation of bronchial plastics during PB. Therefore, the D‐dimer level also has a certain value as an index for determining MPP and producing PB. The PCT level can reflect the degree of systemic inflammation and is positively correlated with the level of inflammation [24]. After MP infection, monocytes and macrophages are induced to increase PCT secretion, which increases the concentration of PCT, resulting in cell damage and severe immunoinflammatory reactions [25]. A previous clinical study on glucocorticoid pulse therapy for refractory MPP in children showed that the level of PCT was significantly elevated in the group treated with high‐dose methylprednisolone, suggesting a close correlation between PCT and the severity of pneumonia [26], which consistent with the results of the present study.

The ESR is a nonspecific inflammatory indicator that is maintained at a low level in the normal body but is significantly increased when stimulated by the inflammatory response. A severe inflammatory response and bronchial plastic formation in PB children can stimulate the ESR to increase. In this study, there were significant differences in the PCT level and ESR between the two groups, and logistic regression analysis revealed that PCT ≥ 0.19 μg/L and ESR ≥ 25.20 mm/h were independent risk factors for MPP combined with PB. This finding is different from the risk factors for MPP and PB reported in another paper, which included CRP and D‐dimer levels [6].

In terms of imaging indicators, the results of this study showed that the proportion of pulmonary consolidation in the PB group was significantly higher than that in the non‐PB group. Pulmonary consolidation refers to a pathological state of the lungs where, due to inflammation, infection, or other causes, the alveoli are filled with inflammatory exudates, blood, or other fluids that replace normal gas, resulting in hardening of the lung tissue texture, which is one of the important indicators for judging the severity of pulmonary infection. This result was consistent with the previous findings, collectively indicating a more intense inflammatory response in the PB group. In a study on the diagnostic model of obliterative bronchiolitis caused by MPP, researchers pointed out that a consolidation range exceeding 2/3 of a lung lobe is an independent risk factor for obliterative bronchiolitis caused by MPP [27], which indirectly illustrates the diagnostic value of pulmonary consolidation in MPP‐related complications. In recent years, with the rapid advancement of machine learning technology, radiomics technology, derived from medical imaging images, has demonstrated crucial application value in the differential diagnosis and prognosis prediction of various diseases [28]. However, there are currently no relevant research reports on the application of radiomics technology in the risk prediction of MPP complicated with PB. This direction may become one of the research entry points with important exploratory significance in this field in the future.

In summary, this study showed that an ESR ≥ 25.20 mm/h and PCT ≥ 0.19 μg/L can be used as predictors of MPP complicated with PB, and monitoring the above indicators in MPP patients can help clinicians identify and diagnose PB early. Bronchial plastics of children with MPP complicated with PB contain a large amount of fibrin, which may be related to the abnormal activation of coagulation and fibrinolysis systems caused by the inflammatory response, but this finding needs to be verified by further studies.

As a single‐center retrospective study, this research has a limited sample size (a total of 75 cases, including 21 cases in the PB group and 54 cases in the non‐PB group). This may lead to the results being influenced by the single‐center diagnosis and treatment process as well as the regional characteristics of patients, resulting in limitations when extrapolating the findings to other regions or medical institutions. Therefore, this study only identified relevant risk factors and did not construct a related prediction model. In the future, multicenter prospective studies can be conducted to expand the sample size and include children with MPP from different regions and hospitals of various levels, so as to improve the external validity of the results. Meanwhile, radiomics technology can be integrated to build multimodal and multidimensional prediction models, further optimize the risk stratification and early intervention protocols for MPP complicated with PB, and promote the translation of research findings into clinical practice.

## Author Contributions

Jian‐Min Dong wrote the manuscript, Chun‐Qing Zhou collected the data, and Yuan‐Yuan Zhen guided the study. All authors reviewed, edited, and approved the final manuscript and revised it critically for important intellectual content, gave final approval of the version to be published, and agreed to be accountable for all aspects of the work.

## Funding

The authors received no specific funding for this work.

## Ethics Statement

This study was approved by the Medical Research Ethics Committee of our hospital.

## Conflicts of Interest

The authors declare no conflicts of interest.

## Data Availability

The data that support the findings of this study are available from the corresponding author upon reasonable request.
